# Parts-per-quadrillion level gas molecule detection: CO-LITES sensing

**DOI:** 10.1038/s41377-025-01864-4

**Published:** 2025-04-30

**Authors:** Haiyue Sun, Shunda Qiao, Ying He, Xiaorong Sun, Yufei Ma

**Affiliations:** 1 National Key Laboratory of Laser Spatial Information, Harbin Institute of Technology, Harbin, 150001 China; 2 Zhengzhou Research Institute, Harbin Institute of Technology, Zhengzhou, 450000 China

**Keywords:** Optical spectroscopy, Infrared spectroscopy

## Abstract

Highly sensitive gas detection plays a crucial role in advanced scientific and technological fields. This paper presents a parts-per-quadrillion (ppq) level ultra-highly sensitive light-induced thermoelectric spectroscopy (LITES) sensor for the first time. The artificial fish swarm algorithm auto-designed multi-pass cell (MPC) with double helix pattern, and the polymer modified round-head quartz tuning fork (QTF) with low-resonant frequency (*f*_0_) were adopted to improve the gas absorption and QTF’s detection ability. The obtained MPC, with a long optical path length (OPL) of 25.8 m and a small volume of 165.8 ml, is beneficial for increasing gas absorption while keeping the sensor compact. The novel QTF was structurally optimized to obtain low *f*_0_ (~9.5 kHz) and modified by polydimethylsiloxane (PDMS) to reduce heat diffusion and enhance vibration amplitude. A strong absorption line of carbon monoxide (CO) located in the mid-infrared region (4.59 μm) was chosen as the target line. The signal-to-noise ratio (SNR) of CO-LITES sensor based on the novel QTF was improved by 10.59 times, reaching the highest level when compared to the commercial QTF. The corresponding minimum detection limit (MDL) was calculated to be 23 ppt. When the integration time of the sensor system was increased to 500 s, the MDL could be improved to 920.7 ppq. Compared to the reported spectroscopy techniques for CO gas detection, the LITES sensor in this study offers an excellent result in terms of detection sensitivity.

## Introduction

Highly sensitive gas detection is crucial in many advanced scientific and technological fields^1–8^, such as semiconductor manufacturing, energy innovation, and interplanetary exploration. In the production of semiconductor chips, trace impurities within electron gases can significantly impact the chip’s yield and reliability^9^. As for energy innovation, hydrogen (H_2_) fuel cell, an emerging environmentally friendly energy source, has garnered substantial interest. Nonetheless, the propensity of H_2_ to incorporate trace carbon monoxide (CO) by-product poses a threat of poisoning the fuel cell^10^. The presence of methane (CH_4_) on Mars is another focal point of international research, with its ultra-low concentration presenting a substantial challenge for detection^11^. These instances underscore the paramount importance of ultra-highly sensitive gas detection techniques.

Laser absorption spectroscopy (LAS) has gained significant attention in recent years due to the rapid response and high sensitivity^12–20^. Among the various techniques in this field, quartz-enhanced photoacoustic spectroscopy (QEPAS) has garnered significant attention due to its advantages of small size, low cost, and high Q factor since it was first reported in 2002^21^. However, a notable drawback of QEPAS is that the quartz tuning fork (QTF) must be submerged in the gas environment. This becomes problematic when dealing with acidic or corrosive gases, such as hydrogen chloride, as the silver electrode on the QTF’s surface can undergo corrosion after prolonged exposure^22,23^. Furthermore, the short absorption path in this technique restricts further enhancement of detection capability. To address these problems, light-induced thermoelastic spectroscopy (LITES) was presented in 2018^24^. LITES sensor inverts gas information from thermoelectric signal of QTF, which is stimulated by laser passing through the gas. Therefore, the LITES sensor, as a non-contact sensing technology, can effectively prevent the QTF from being corroded by acidic or corrosive gases. In addition, it can also further improve the detection capability by increasing the gas absorption path.

The sensitivity enhancement of LITES technology is primarily constrained by two aspects: the optical absorption intensity and the characters of QTF. According to the Beer-Lambert’s law, increasing optical path length (OPL) can availably improve the absorption intensity^25–28^. Multi-pass cell (MPC) is a widely used method to increase the effective OPL^29–32^. However, longer OPL of MPC often requires a larger volume (V), which is not conducive to system integration. Therefore, OPL/V was presented to comprehensively assess the performance of MPC. Finding a balance between OPL and V, and achieving longer OPL within more compact system, are current challenges in MPC design. Regarding the performance of QTF, QTFs currently used in LITES are mostly designed for commercial crystal oscillators in chips, with a high resonant frequency (*f*_0_) of 32.768 kHz^33–37^. A high *f*_0_ leads to a low energy accumulation of QTF, which reduces sensing signal amplitude. Therefore, some research teams have conducted studies on novel QTF with low *f*_0_^38–42^. However, these new reported QTFs face two issues: firstly, the QTF’s head is often designed to be straight, which limits further enhancement of the stress degree during vibration. Additionally, the reported QTFs with low frequency mostly use silver or gold as electrodes, which have high effective thermal conductivities (*Φ*) resulting a massive diffusion waste of heat. In addition, their low coefficient of thermal expansion (*β*) decrease the vibration amplitudes of the QTFs. All of these issues reduce the conversion efficiency of the thermal signal to the electrical signal in LITES sensor.

In this paper, we present a parts-per-quadrillion (ppq) level LITES sensor, for the first time, based on intelligent algorithm optimized MPC with double helix pattern and polymer modified low frequency QTF with round-head. Artificial fish swarm algorithm (AFSA) was employed to iteratively optimize the optical model of MPC via three mirrors, resulting in a double helix spot pattern through astigmatism. The OPL/V of the novel MPC is up to 15.6 cm^−^^2^ (25.8 m/165.8 ml), which means that a highly dense pattern of spot distribution is on the mirrors and the system is compact. The QTF designed by finite element analysis has characters of round-head and low *f*_0_ (~9.5 kHz). Polydimethylsiloxane (PDMS) on surface of the novel QTF was used to reduce heat diffusion and increase the stress during vibration. CO molecule was selected to verify the sensing performance, and a distributed feedback quantum cascade laser (DFB-QCL) with emission wavelength at 4.59 μm was selected as the light source.

## Results

### The intelligent algorithm auto-designed a three-mirror MPC with a double helix pattern

**Fig. 1:**
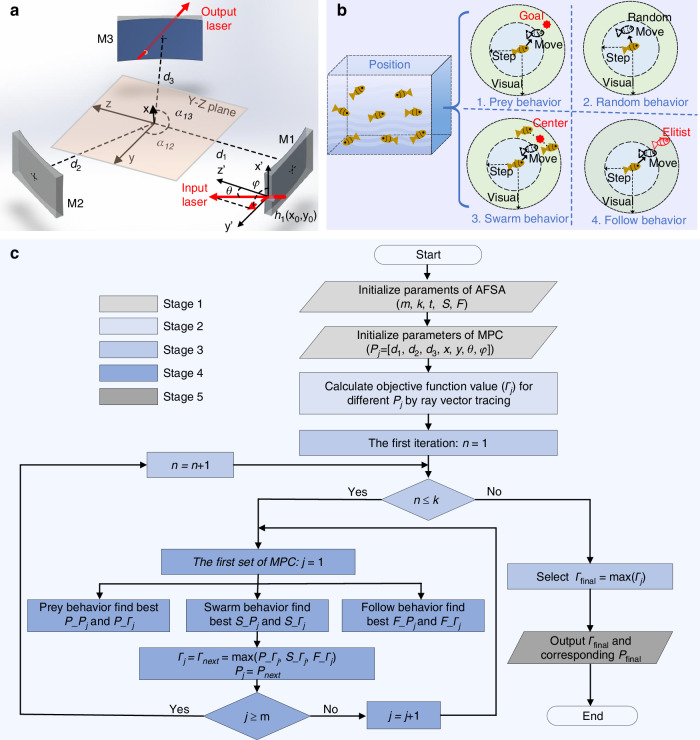
**Theoretical model of MPC design.** **a** Structure model of three-mirror MPC. *d*: distance between mirror and origin. *α*: angle between mirrors; *θ*: the angle between the incident laser and the *z* axis; *φ*: the angle between the projection of the incident ray in the x-y plane and the *x* axis. *h*_1_(*x*_0_, *y*_0_): coordinates of laser entry hole. **b** Theoretical model of ASFA design algorithm. Position: *P* = [*d*_1_, *d*_2_, *d*_3_, *x*_0_, *y*_0_, *θ*, *φ*], seven-dimensional position information for fish swarm. **c** The algorithm flow chart of MPC design. *P*_*j*_ is the multidimensional coordinate of *j*_th_ fish. *Γ*_*j*_ is the corresponding objective function value. *m*, *k,* and *t* represent the number of fish, algorithm iterations, and try work, respectively. *S* and *F* represent the step size and visual field of the artificial fish, respectively

Two-mirror Herriott MPC is limited in its ability to form circular or elliptical spot patterns without a central distribution, which precludes the realization of a compact design with a long OPL. To overcome this limitation, an additional mirror was incorporated into the two-mirror cavity to induce astigmatism and generate dense spot pattern. The complex optical structure is better suited for vector ray tracing simulation. However, manual parameter searching in this simulation lacks precision and fails to fully exploit the potential of three-mirror MPCs. AFSA is renowned for its rapid convergence, high robustness, and excellent global search capabilities, making it highly suitable for solving complex optimization problems. In this research, AFSA was first employed to automatically determine the structural parameters of a high-performance three-mirror MPC. The structure model of three-mirror MPC shown in Fig. 1a is consisted of three spherical mirrors with same curvature (*R*) of 100 mm. The mirrors’ centers are all put into y-z plane and around *x* axis. The direction from the M1 center to the origin is defined as the z axis. Distances from mirrors to the origin and angle between mirrors are presented by *d* (*d*_1_, *d*_2_, and *d*_3_) and *α* (*α*_12_ = *α*_13_ = 120°), respectively. The incident light entering through hole 1 (*h*_1_) on M1 is reflected by M2, M3, and M1 in turn to complete a ring path. The position of *h*_1_ and angle of the incident laser could be determined by (*x*_0_, *y*_0_) and (*θ*, *φ*), respectively. Figure 1b shows the principle of AFSA, simulating the prey, swarm, follow, and random behaviors of the fish swarm to search for the best position with the highest food concentration.

In the auto-design process of MPC, the number of fishes in fish swarm is presented by *m*, and the optical system could be determined by the multidimensional coordinate (*P*_*j*_) selected as the fish position:

1 $${P}_{j}=[{d}_{1},{d}_{2},{d}_{3},{x}_{0},{y}_{0},\theta ,\varphi ],j=1,2,{..}.,m$$

and the food concentration could be presented by the objective function value (*Γ*):

2 $${\varGamma} ={\omega }_{1}\cdot {\rm{OPL}}+{\omega }_{2}\cdot {\rm{OPL}}/{\rm{V}}$$

where *ω*_1_ and *ω*_2_ are the weight factors used to balance the values between OPL and OPL/V. Among them, OPL could be obtained by vector ray tracing:

3 $${\rm{OPL}}=\mathop{\sum }\limits_{i=1}^{i=N-1}{\rho }_{i}=\mathop{\sum }\limits_{i=1}^{i=N-1}(\sqrt{{({n}_{i}\cdot ({M}_{i}-{r}_{i}))}^{2}-({M}_{i}-{r}_{i})\cdot ({M}_{i}-{r}_{i})+{R}^{2}}-{n}_{i}\cdot ({M}_{i}-{r}_{i}))$$

4 $${n}_{i}=\frac{{M}_{i}-{r}_{i}}{R}$$

5 $${M}_{(i+1)(i+2)}={M}_{i(i+1)}-2\cdot ({M}_{i(i+1)}\cdot {n}_{i})\cdot {n}_{i}$$

6 $${M}_{i+1}={M}_{{\rm{i}}}+{\rho }_{{\rm{i}}}\cdot {M}_{i(i+1)}$$

where *M*_i_ represents the coordinate of the *i*_th_ (*i* = 1, 2…*N*) spot. *M*_*i*(*i+*1)_ and *ρ*_*i*_ represent the normal direction vector and length of the *i*_th_ ray, respectively. *n*_*i*_ and *r*_*i*_ represent the normal vector and corresponding sphere center, respectively. The design algorithm flow chart is shown in Fig. 1c. The parameters of the fish swarm should be initialized first. In the iterative process, prey, swarm, and follow behaviors are combined with the ray vector tracing to optimize *P*_*j*_ and corresponding *Γ*_*j*_. Random behavior unlabeled is taken as the replacement behavior.

**Fig. 2:**
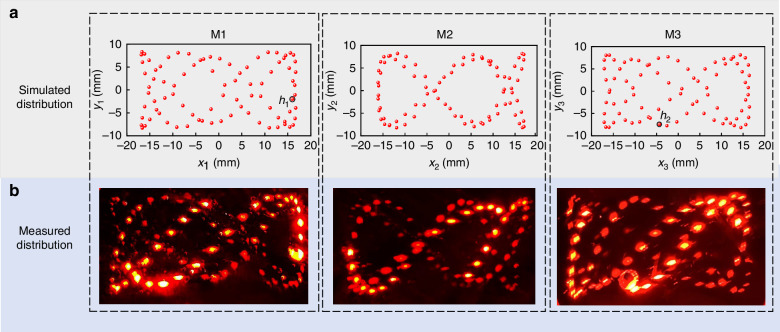
**Double helix pattern of three-mirror MPC.** **a** Simulated distribution of double helix pattern. **b** Measured distribution of double helix pattern

The mirrors were all silver-coated with reflectivity of 98%, offering low cost and broad wavelength applicability. In LITES technology, laser power impacts signal amplitude of QTF. Consequently, the times of reflections (*γ*) were limited within 262 to ensure a transmittance exceeding 5%. The structural parameters were optimized by the intelligent algorithm and are shown in Table 1. When *γ* reached up to 260, the light passed through *h*_1_. To adjust a reasonable placement of the detector and laser, the exit hole was set on the M2 mirror with *γ* of 259. The real distribution of light spots obtained with red diode lasers is shown in Fig. 2. The light spots have a double helix distribution, and this dense spot pattern can improve OPL/V more effectively than the widely used Herriott MPC’s ring or oval spot pattern.

**Table 1:** Parameters of three-mirror MPC

Variables	*d*_1_, *d*_2_, *d*_3_ (mm)	*θ*, *φ* (°)	*x*_0_, *y*_0_ (mm)	*N*	OPL (m)	V (ml)	OPL/V (cm^−2^)
Results	59, 57, 57.4	17.2, 106.7	-2, 16	259	25.8	165.8	15.6

The OPL, V and OPL/V ratio of the novel MPC were determined as 25.8 m, 165.8 ml, and 15.6 cm^−2^, respectively. The specifications for the other currently reported MPCs are shown in Table 2. It can be seen that the auto-designed three-mirror MPC with a double helix pattern has more reflections of light in a compact structure. The higher OPL/V of the MPC is conducive to improving detection sensitivity and maintaining a compact sensor design.

**Table 2:** Parameters comparison among different reported MPCs

Pattern types	*γ*	OPL (m)	V (mL)	Total OPL/V (cm^−2^)
Nine-circle spot pattern^47^	235	32.7	281.7	11.6
Petal spot pattern^48^	183	20.4	332.1	6.14
Toroidal MPC^49^	51	4.1	40	10.3
Segmented circular MPC^50^	64	9.9	140	7
Double helix pattern MPC (this paper)	259	25.8	165.8	15.6

### The polymer-modified round-head QTF with low *f*_0_

Reducing resonant frequency *f*_0_ of the QTF is conducive to increasing the energy accumulation time, thereby improving the detection sensitivity of the sensor. The *f*_0_ of the QTF is given by the following formula^43^:

7 $${f}_{0}=\frac{\pi T}{8\sqrt{12}{L}^{2}}\sqrt{\frac{E}{\eta }}{\chi }^{2}$$

where *E* and *η* are the modulus of elasticity and density of the QTF, respectively. *χ* is the number of modes. *T* and *L* are the thickness and length of the fork fingers, respectively. From the formula, it can be concluded that *f*_0_ can be reduced by increasing *L* and reducing *T*. In this study, a finite element model of the QTF was established to optimize the structural parameters. Compared with commercial QTF, *L* of the designed QTF increased from 3.9 mm to 9.1 mm, and *W* decreased from 0.36 mm to 0.25 mm. In addition, two round heads were added to the top of the QTF to enhance the stress during vibration. Gold electrode was selected to improve the oxidation and corrosion resistances.

**Fig. 3:**
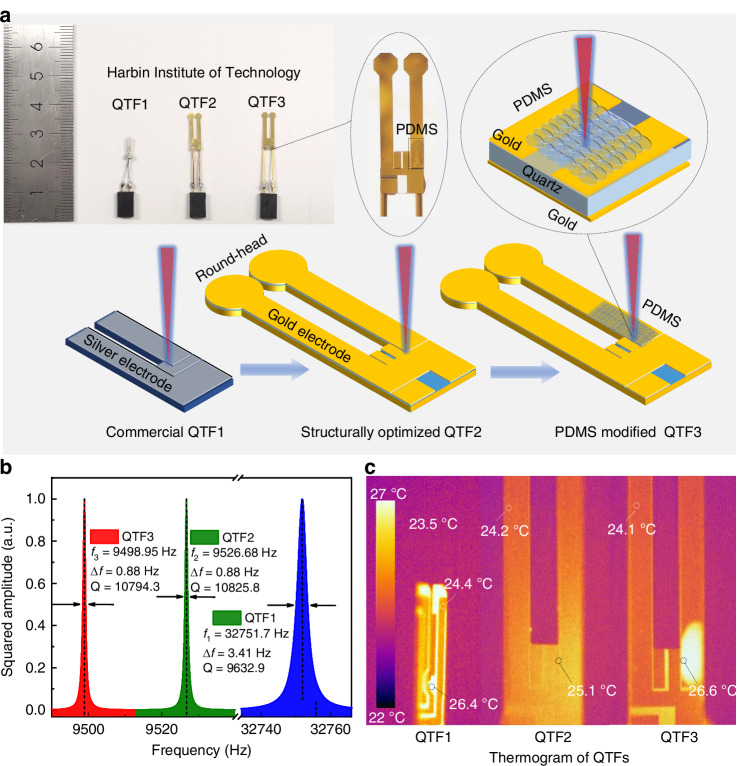
**The polymer modified round-head QTF with low*f***_0_. **a** Schematic diagram of QTF optimization. Insert: real picture of different QTFs. QTF1: commercial QTF with high *f*_0_. QFT2: structurally optimized QTF with low frequency and round head. QFT3: PDMS modified low-frequency QTF with P-Q-G sandwich structure. PDMS polydimethylsiloxane, a polymer material with low *Φ* and high *β*. **b** Frequency responses of different QTFs. **c** Thermogram of different QTF

In addition to the structural geometric parameters, the vibration amplitude (*δ*) of QTF has also been affected by the material properties, as expressed by the following formula^44^:

8 $$\delta \propto \frac{({\beta }_{1}-{\beta }_{2})\cdot L}{{\varPhi }_{1}{T}_{1}+{\varPhi }_{2}{T}_{2}}$$

where *Φ*_1_ and *Φ*_2_ are the effective thermal conductivities of the electrode and quartz, respectively. *β*_1_ and *β*_2_ are the thermal expansion coefficients of the electrode and quartz, respectively. Based on the formula, *δ* can be increased by decreasing *Φ*_1_ and increasing *β*_1_. However, the gold electrode has high *Φ*_1_ (317 W m^−^^1^ k^−^^1^) and low *β*_1_ (14.2 × 10^−^^6^ k^−^^1^), which is not conducive to improving the signal amplitude. In order to solve this problem, as shown in Fig. 3a, the wet-etched slit of the gold electrode at the QTF’s root and near QTF’s finger was coated with PDMS. The laser was focused on the sandwich structure made of PSMS-Quartz-Gold (P-Q-G). PDMS is a polymer material with low thermal conductivity (*Φ*_3_ = 0.18 W m^−^^1^ k^−^^1^) and high thermal expansion coefficient (*β*_3_ = 960 × 10^−^^6^ k^−^^1^). The potential coating materials for QTF mainly include PDMS, Polyimide (PI), graphene, and carbon nanotubes. PI has excellent chemical stability and can withstand high temperatures, strong acids, strong bases, and the erosion of most organic solvents. Therefore, it is more suitable for gas sensing in harsh chemical environments. However, its thermal expansion coefficient is relatively low (~30 × 10^−^^6^ k^−^^1^), which is not conducive to enhancing the vibration amplitude of the QTF. As for graphene and carbon nanotubes, they have strong thermal diffusion capabilities (greater than 3000 W m^−^^1^ k^−^^1^), which is not conducive to heat accumulation. In contrast, PDMS with low effective thermal conductivity and high thermal expansion coefficient can effectively increase the local temperature and stress of vibration, which can improve the detection sensitivity of the sensor.

As shown in Fig. 3b, the *f*_0_ of QTF1, QTF2, and QTF3 were measured to be 32751.7 Hz, 9526.68 Hz, and 9498.95 Hz, respectively. Compared with the commercial QTF1, the structurally optimized QTF2 can effectively reduce *f*_0_ by 70%, which is conducive to longer energy accumulation times. If *f*_0_ is further reduced, it is likely to introduce ambient low-frequency noise. In addition, compared with commercial QTF, thickness of the structurally optimized QTF2 decreased from 0.36 mm to 0.25 mm, enabling it to have a higher quality factor (*Q* = 10825.8). The thermogram of different QTFs under laser irradiation were captured and are shown in Fig. 3c. The output power of the laser power was set to 20 mW and the ambient temperature was 23.5 °C. The energy exchanges of gold and silver with the external environment are both faster than quartz, so the temperature of the electrode is relatively lower. Near the focus positions presented in Fig. 3a, the maximum temperatures of the three QTFs were 26.4 °C, 25.1 °C, and 26.6 °C, respectively. Due to the larger size, the temperature of structurally QTF2 was lower than QTF1. As for QTF3, the PDMS reduced the diffusion of heat, and the local temperature was obviously increased.

### CO-LITES sensor based on the optimized MPC and QTF

**Fig. 4:**
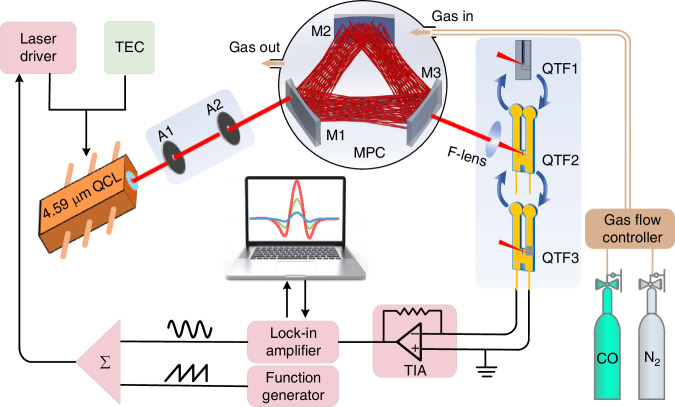
**Schematic diagram of CO-LITES sensor based on intelligent algorithm optimized MPC with double helix pattern and PDMS modified round-head QTF with low*f***_0_. F-lens focusing lens, TIA transimpedance amplifier, A1 and A2 two apertures used to determine the incident angle of the laser beam

The schematic diagram of the mid-infrared CO-LITES sensor based on the optimized MPC and QTF is shown in Fig. 4. In this study, the strong mid-infrared absorption line of CO located at 4587.64 nm (2179.77 cm^−1^) was chosen as the target line. A DFB-QCL with a central wavelength of 4.59 μm was used as the excitation source. The TEC temperature of the laser was set to 35 °C. When the input current was 301 mA, the output wavelength of the laser could match the selected absorption line with an output power of 145 mW. A relatively high laser output power can significantly improve the detection sensitivity of the system. The absorption line strength is 4.079 × 10^−19^ cm^−1^/(molecule·cm^−^^2^), which is conducive to enhancing the absorption of the laser by the gas. The laser passed through two apertures (A_1_ and A_2_) and then was incident into optimized MPC with dense double helix spot pattern. Position parameters of apertures and mirrors in MPC was adjusted by He-Ne laser. After 259 times reflections by three mirrors, laser exited from MPC and then was focused on different QTFs to compare their sensor performances. The focus position was chosen at the slit of the electrode at the QTF’s root. PDMS was used to modify QTF3 to reduce heat diffusion and enhance thermal expansion coefficient. The focal length of focusing lens (F-lens) was 8 mm. The small focal length is beneficial to reduce the focal spot radius and improve the local temperature gradient on the QTF. Vibration signal generated by thermal expansion of QTF is converted into an electrical signal based on its piezoelectric effect. Wavelength modulation spectroscopy (WMS) was adopted in this system to suppress the background noise. Ramp and sine waves were utilized to modulate the laser source. The sine waves’ frequencies were set to 16375.85 Hz, 4763.34 Hz, and 4749.48 Hz, matching the *f*_0_/_2_ of QTF1, QTF2, and QTF3, respectively. Electrical signal from QTF was demodulated by a lock-in amplifier to get the second harmonic signal (2 *f*). The corresponding integration time was set to 20 ms. The concentration of CO within MPC was precisely manipulated by fine-tuning the flow rates of 1 ppm CO standard gas and ultra-pure nitrogen (N_2_).

**Fig. 5:**
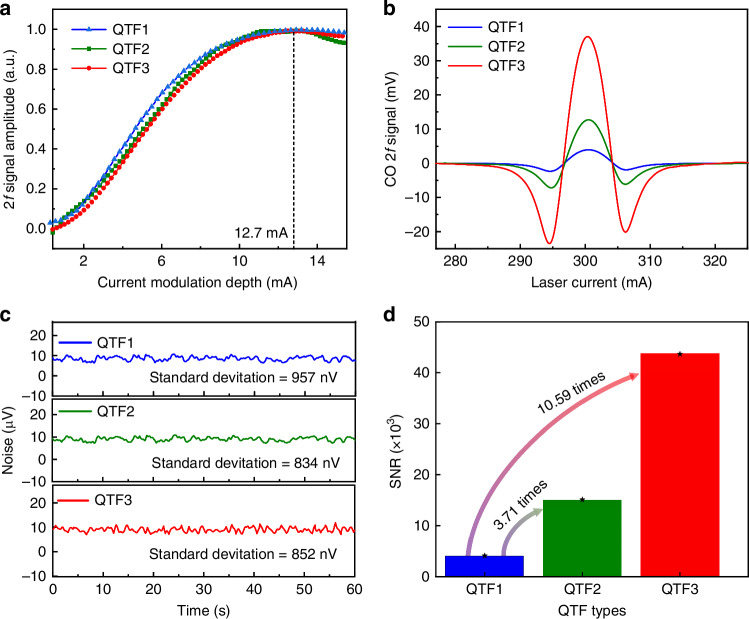
**Performance comparison of the CO-LITES sensor with the three different QTFs.** **a** The relationships between the 2 *f* signal amplitude and the current modulation depth of different QTFs. **b** 2 *f* signal of different QTFs. **c** Noise level of the sensor system. **d** Calculated SNR results of different QTFs

In WMS technique, modulation depth is an important parameter influencing the signal amplitude. The relationship between the 2 *f* signal amplitude and the current modulation depth of different QTFs was measured and is shown in Fig. 5a. It can be seen that as the current modulation depth increases, the signal first increases and then slowly decreases. The maximum 2 *f* signal amplitude can be obtained when the current modulation depth is 12.7 mA. Consequently, this value was used in the subsequent experiments. The corresponding 2 *f* signals for 1 ppm CO based on optimized MPC and different QTFs are shown in Fig. 5b. The 2 *f* signals of QTF1, QTF2, and QTF3 were 3.93 mV, 12.69 mV, and 37.05 mV, respectively. The corresponding noises shown in Fig. 5c were 957 nV, 834 nV, and 852 nV, respectively. Based on the above results, the signal-to-noise ratios (SNRs) of the three QTFs were calculated to be 4106.58, 15215.83, and 43485.92, respectively. As shown in Fig. 5d, compared to the commercial QTF1, the SNR of optimized QTFs was obviously improved. The increased factor of structurally optimized QTF2 was 3.71 times, and the increased factor of PDMS modified QTF3 with P-Q-G sandwich structure was 10.59 times, reaching the highest level when compared to the commercial QTF in the reported LITES system. This significant improvement can be attributed, on one hand, to the structural optimization, which has extended energy accumulation time and increased stress during vibration. On the other hand, it benefits from the P-Q-G sandwich structure that has mitigated heat diffusion and ameliorated the thermal expansion coefficient. The minimum detection limit (MDL), defined as the ratio of gas concentration (C) to the SNR^45^, MDL = C/SNR, was calculated to be 23 ppt for this CO-LITES sensor based on QTF3. This demonstrated the great potential of QTF3 in achieving ultra-highly sensitive gas detection.

**Fig. 6:**
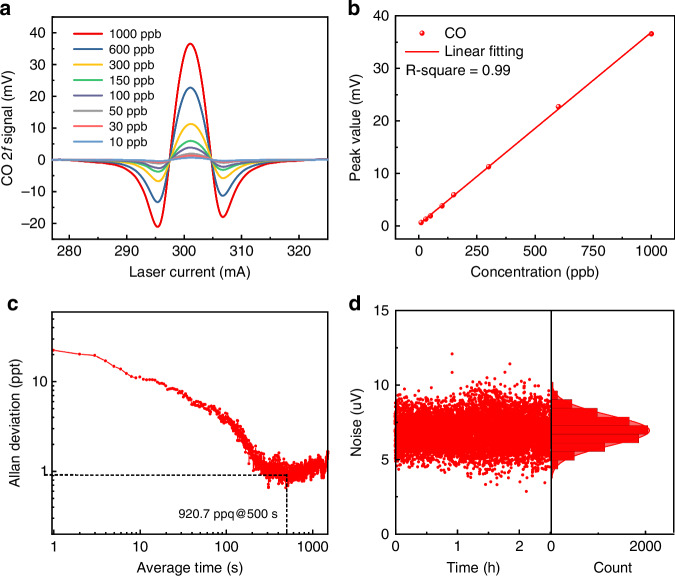
**Performance of CO-LITES sensor based on optimized MPC and QTF3.** **a** 2 *f* signal of the CO-LITES sensor with different CO concentrations. **b** The linear relationship between 2 *f* signal amplitude and CH_4_ concentration. **c** Allan variance analysis for CO-LITES sensor. **d** Continuous noise detection in the sensor and corresponding normal distribution

The relationship between CO concentration and 2 *f* signal of CO-LITES sensor based on optimized MPC and modified QTF3 was investigated and is shown in Fig. 6a, b. Different concentrations of CO were obtained by mixing 1 ppm CO and ultra-pure N_2_. The experimental results indicated that the signals of CO-LITES sensor were directly proportional to their respective concentrations. The high *R*^2^ of 0.99 for the linear fitting indicates the CO-LITES sensor had an excellent linear response to ultra-low CO concentration. Allan deviation analysis was used to investigate the long-term stability of the system and evaluate the optimal detection performance^46^. Ultra-pure N_2_ was flushed into the three-mirror MPC. As depicted in Fig. 6c, d, by adjusting the optimized integration time to 500 s, the MDL of the reported CO-LITES sensor was enhanced to a record result of 920.7 ppt.

In this CO-LITES sensor, the mid-infrared QCL was combined with the intelligently optimized three-mirror MPC to greatly enhance the absorption of CO in a compact system. Compared to the commercial QTF, a structurally optimized QTF with a round head and low *f*_0_ was modified by PDMS, for the first time, to obtain the large amplitude improvement in this LITES sensor. For the above reasons, this study achieved the highest detection sensitivity of gas molecules in all reported LITES sensors. A comprehensive comparison with the current reported spectroscopy technique for CO gas detection is presented in Table 3. It can be observed from the comparison results that the novel LITES sensor proposed in this research possesses a distinct advantage in terms of high sensitivity. This holds promise for applying the technology to cutting-edge scientific fields that demand exceptionally high detection sensitivity.

**Table 3:** Comparison of CO detection among different spectroscopy techniques

Sensor type	Fundamental	Wavelength	Output power	Special method	Detector	MDL
CO-PAS^51^	Photoacoustic	1.57 μm	10 W	Dual-resonator PAC	Microphone	342.7 ppb
CO-QEPAS^52^	Photoacoustic	4.57 μm	32.3 mW	Water-promotion	T-shaped QTF	12 ppb
CO-TDLAS^53^	Laser absorption	4.54 μm	—	Improved White cell	Photodetector	133 ppt
CO-ICOS^54^	Cavity-enhanced absorption	4.88 μm	55 mW	Off-axis integrated cavity	Photodetector	3 ppb
This paper	Light-induced thermoelastic	4.59 μm	145 mW	Double helix pattern MPC	PDMS modified QTF with novel structure	920.7 ppq

*ppb* 10^−9^, *ppt* 10^−12^, *ppq* 10^−15^

**Fig. 7:**
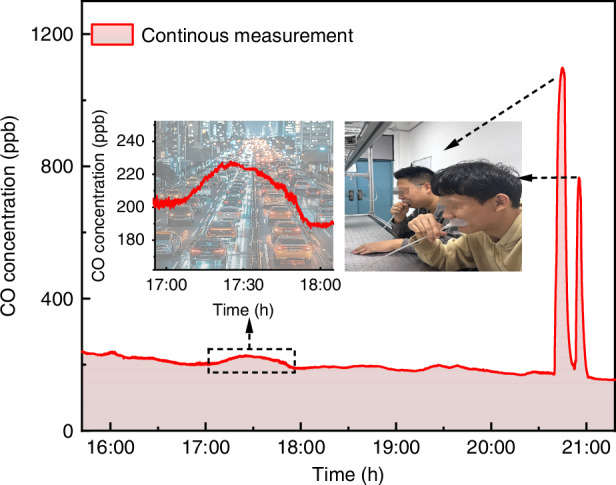
Examples of CO measurement applications. CO concentration measurement on campus and human breath using this CO-LITES sensor

Additionally, examples of applications in urban environmental monitoring and human health detection were provided. The CO concentration on the campus of Harbin Institute of Technology was continuously monitored on 19 September 2024, from dusk to dawn, by this CO-LITES sensor. A vacuum pump was used to draw external gases into the MPC at a flow rate of 500 sccm. As shown in Fig. 7, as human activity decreased late at night, the CO concentration in the air also decreased. However, there was a brief increase in gas concentration after 17:00, as it was the evening rush hour in the city and there was a large volume of traffic. After that, the CO concentration in human breath was tested by collecting the concentration of exhaled gas through a breath collection bag and then drawing it into the MPC at the same flow rate. As a trace by-product of human metabolism, CO can be effectively used as one of the evaluation parameters of human lung health.

## Discussion

In this paper, a ppq-level CO-LITES sensor was demonstrated for the first time, as far as we know, to realize ultra-highly sensitive gas detection. The sensor integrated two key optimized components: an intelligent algorithm auto-designed three-mirror MPC and a polymer-modified round-head QTF. Specifically, the AFSA was utilized to optimize the optical model of a three-mirror MPC. This optimization led to the formation of a double helix pattern with 259 high reflection times. The resulting MPC featured a long OPL of 25.8 m and a small volume of 165.8 ml. This method is beneficial for enhancing gas absorption while keeping the sensor compact. Regarding the QTF, the self-designed round-head QTF was structurally optimized to reduce the resonant frequency to ~9.5 kHz, significantly improving the energy accumulation time. Additionally, the newly designed QTF was further modified with PDMS, which has a low thermal expansion coefficient and high effective thermal conductivity, to reduce heat diffusion and increase the stress on the QTF. A strong absorption line of CO located in the mid-infrared region was chosen as the target line. The SNR of the CO-LITES sensor, based on the PDMS modified round-head QTF, was improved by 10.59 times compared to the commercial QTF. The MDL of this CO-LITES sensor was determined to be 23 ppt. When the integration time of the sensor system was increased to 500 s, the MDL could be improved to 920.7 ppq. Finally, this paper presented examples of CO measurement applications in urban environmental monitoring and human health assessment. In addition, utilizing high-reflectivity dielectric-coated mirrors, vacuum-encapsulated QTF, and algorithmic noise reduction could further enhance the sensor’s performance.

## Materials and methods

### Build of the three-mirror MPC with a double helix pattern

The three-mirror MPC consisted of three spherical mirrors in y-z plane with the same curvature of 100 mm. The mirrors were cut into the same rectangle with side lengths of 20 mm and 40 mm. Reflectivity of silver coating on mirrors was 98%, offering low cost and broad wavelength applicability. According to the design result, distances from M1, M2, and M3 to the origin were 59 mm, 57 mm, and 57.4 mm, respectively. The angle between adjacent mirrors was 120°. The incident light entered through the *h*_1_ on M1 and exited through *h*_2_ on M3. The diameters of *h*_1_ and *h*_2_ were 2 mm and 3 mm, respectively. The small diameter of *h*_1_ helps to reduce the diameter of the light spot and avoid the laser interference. The thickness of the mirror was 4 mm. He-Ne laser was used as an indicator laser to adjust the optical system path. To optimize the adjustment process, we have adopted an integrally machined installation platform equipped with guide rails. The mirrors were placed on the machined mirror mounts, which were connected to the installation platform. Adjusting the mirrors along the guide rails allowed us to reduce the original multiple dimensions of adjustment to a single dimension. The tolerance for the positional accuracy of the guide rails could be maintained within ±0.2 mm. Position parameters of apertures determined by He-Ne laser were used to adjust the direction of the mid-infrared detecting laser. To verify that the actual light spot pattern coincides with the theoretical pattern, the OPL of the MPC was tested through direct absorption spectroscopy. The deviation between the measured OPL and the theoretical one was negligible, and it was only 1.94%.

### Preparation of the polymer-modified round-head QTF

In this study, a finite element model of the QTF was established to optimize the structural parameters. Compared with commercial QTF, *L* of the designed QTF increased from 3.9 mm to 9.1 mm, and *W* decreased from 0.36 mm to 0.25 mm. The optimization of these parameters is conducive to reducing *f*_0_ of the QTF. Lower frequency of the QTF enabled longer energy accumulation times, thus augmenting the piezoelectric conversion performance. It is also noteworthy that the large round head of the novel QTF enhances the overall center of gravity, increasing the moment of force during vibration. A gold electrode was selected to improve the oxidation and corrosion resistance. Both of these aspects contribute to an increase in the deformation and surface charge density of the QTF. After the design was completed, the actual processing of the QTF began. The cleaned quartz wafer was evenly coated with photoresist, and a mask was used for photolithographic exposure. Post-exposure was carried out to reveal the QTF pattern. Next, wet etching with etching solutions was adopted to remove the unprotected quartz material and form the QTF’s structure. The dimensional tolerance ranges of the QTF can be controlled within ±3 μm. Finally, gold was chosen as the electrode material and was deposited at specific positions on the QTF via physical vapor deposition technology. The wet-etched slit of the gold electrode at the QTF’s root and near QTF’s finger was coated with PDMS to reduce heat diffusion and enhance vibration amplitude. The thermograms of different QTFs under laser irradiation were captured by a thermal infrared imager (model No. Fotric 246 M, Fotric Inc.)

### Build of the CO-LITES sensor system

A mid-infrared DFB-QCL was used as the excitation source. Full beam waist and M^2^ factor of the laser were 2.36 mm and 1.19, respectively. The TEC temperature of the laser was set to 35°C. When the input current was 301 mA, the output wavelength of laser could match the selected absorption line at 4587.64 nm (2179.77 cm^−1^) with output power of 145 mW. The laser diver (model No. QCL 1500 LAB) and temperature controller (model No. TC 10 LAB) were supplied by Wavelength Electronics Inc. The laser passed through two apertures and then was incident into the MPC from M1. The diameters of the apertures in the optical system were 2 mm. After 259 times of reflections by three mirrors, the laser exited from M3 and was then focused on different QTFs. The focus position was chosen at the slit of the electrode at the QTF’s root. The focal length of the focusing lens was 8 mm. The Vibration signal generated by the thermal expansion of the QTF is converted into an electrical signal. The electrical signal was amplified by a TIA and input into a phase-locked amplifier (model No. MFLI DC-500 kHz, Zurich Instruments). Ramp and sine waves were utilized to modulate the laser source. The sine waves’ frequencies were set to 16375.85 Hz, 4763.34 Hz, and 4749.48 Hz, matching the *f*_0_/2 of QTF1, QTF2, and QTF3, respectively. The ramp waves’ frequencies were all set to 100 mHz to ensure a balance between measurement speed and precision. The electrical signal from QTF was demodulated by a lock-in amplifier to get the second harmonic signal. The corresponding integration time was set to 20 ms. A gas flow controller (model No. PIPG-MCF102, Shaanxi Yidu Intelligent Technology Co., Ltd.) was used to manipulate the concentration of CO within MPC by fine-tuning the flow rates of 1 ppm CO standard gas and ultra-pure N_2_ gas. Ultra-pure N_2_ gas was employed to avoid interference from impurities. The CO standard gas met the national secondary standard gas requirements, and the difference between the actual concentration and the true concentration was less than 2%. The total gas flow rate was regulated at 300 sccm. Additionally, the experiment was conducted under normal temperature and pressure.

## Data Availability

The data that support the findings of this study are available from the corresponding author upon reasonable request.
